# *Beauveria bassiana* rewires molecular mechanisms related to growth and defense in tomato

**DOI:** 10.1093/jxb/erad148

**Published:** 2023-04-24

**Authors:** Silvia Proietti, Gaia Salvatore Falconieri, Laura Bertini, Alberto Pascale, Elisabetta Bizzarri, Julia Morales-Sanfrutos, Eduard Sabidó, Michelina Ruocco, Maurilia M Monti, Assunta Russo, Kinga Dziurka, Marcello Ceci, Francesco Loreto, Carla Caruso

**Affiliations:** Department of Ecological and Biological Sciences, University of Tuscia, largo dell’Università snc, 01100 Viterbo, Italy; Department of Ecological and Biological Sciences, University of Tuscia, largo dell’Università snc, 01100 Viterbo, Italy; Department of Ecological and Biological Sciences, University of Tuscia, largo dell’Università snc, 01100 Viterbo, Italy; Plant-Microbe Interactions, Department of Biology, Science for Life, Utrecht University, Padualaan 8, 3584 CH Utrecht, Netherlands; Department of Ecological and Biological Sciences, University of Tuscia, largo dell’Università snc, 01100 Viterbo, Italy; Proteomics Unit, Centre de Regulació Genòmica, Barcelona Institute of Science and Technology (BIST), Carrer Dr. Aiguader 88, 08003 Barcelona, Spain; Proteomics Unit, Universitat Pompeu Fabra, Carrer Dr Aiguader 88, 08003 Barcelona, Spain; Proteomics Unit, Centre de Regulació Genòmica, Barcelona Institute of Science and Technology (BIST), Carrer Dr. Aiguader 88, 08003 Barcelona, Spain; Proteomics Unit, Universitat Pompeu Fabra, Carrer Dr Aiguader 88, 08003 Barcelona, Spain; Institute for Sustainable Plant Protection (IPSP-CNR), Piazzale Enrico Fermi, 1, 80055 Portici (NA), Italy; Institute for Sustainable Plant Protection (IPSP-CNR), Piazzale Enrico Fermi, 1, 80055 Portici (NA), Italy; Institute for Sustainable Plant Protection (IPSP-CNR), Piazzale Enrico Fermi, 1, 80055 Portici (NA), Italy; Department of Agricultural Sciences, University of Naples Federico II, Via Università 100, 80055 Portici (NA), Italy; Department of Biotechnology, The Franciszek Górski Institute of Plant Physiology, Polish Academy of Sciences, Niezapominajek 21, 30-239 Kraków, Poland; Department of Ecological and Biological Sciences, University of Tuscia, largo dell’Università snc, 01100 Viterbo, Italy; Department of Biology, Via Cinthia, University of Naples Federico II, 80126, Naples, Italy; Department of Ecological and Biological Sciences, University of Tuscia, largo dell’Università snc, 01100 Viterbo, Italy; Ghent University, Belgium

**Keywords:** *Beauveria bassiana*, *Botrytis cinerea*, hormones, necrotrophic pathogen, oxidative stress, proteome, tomato

## Abstract

Plant roots can exploit beneficial associations with soil-inhabiting microbes, promoting growth and expanding the immune capacity of the host plant. In this work, we aimed to provide new information on changes occurring in tomato interacting with the beneficial fungus *Beauveria bassiana*. The tomato leaf proteome revealed perturbed molecular pathways during the establishment of the plant–fungus relationship. In the early stages of colonization (5–7 d), proteins related to defense responses to the fungus were down-regulated and proteins related to calcium transport were up-regulated. At later time points (12–19 d after colonization), up-regulation of molecular pathways linked to protein/amino acid turnover and to biosynthesis of energy compounds suggests beneficial interaction enhancing plant growth and development. At the later stage, the profile of leaf hormones and related compounds was also investigated, highlighting up-regulation of those related to plant growth and defense. Finally, *B. bassiana* colonization was found to improve plant resistance to *Botrytis cinerea*, impacting plant oxidative damage. Overall, our findings further expand current knowledge on the possible mechanisms underlying the beneficial role of *B. bassiana* in tomato plants.

## Teaser

In tomato, the beneficial fungus *Beauveria bassiana*perturbs key molecular pathways related to primary and secondary metabolism, and growth- and defense-related hormones, and is effective against necrotrophic infection.

## Introduction

The soil–plant interface is a complex and dynamic microenvironment that influences all plant functions. Beneficial associations between plants and microbes have been extensively studied in the microbially dominated environment of the rhizosphere (Zamioudis and Pieterse, 2012; Vandenkoornhuyse *et al.*, 2015; Yu *et al*., 2019; Bakker *et al.*, 2020; Pascale *et al.*, 2020; Saad *et al.*, 2020; Fadiji *et al.*, 2022). These associations are highly advantageous for all organisms involved, as soil microbes can benefit from plant metabolites, while plant growth and development are enhanced by microbes that promote nutrient uptake and/or protection against abiotic stresses. Among soil microbes, fungi provide several examples of evolutionary clades able to build mutualistic interactions with plants. These include endophytic fungi that colonize the endosphere of roots and/or shoots. The highly sophisticated relationship between plants and beneficial endophytic fungi relies on a precise molecular dialogue between the partners, and great efforts have been devoted to untangling this dialogue (Lu *et al.*, 2021). Upon physical contact, endophytic fungi can penetrate the host and successfully colonize plant tissues by employing fungal effectors that control plant defense responses (Lu *et al.*, 2021; Pozo *et al.*, 2021). In addition, several signaling components appear to allow plants to finely regulate their relationship with the fungus. Specific calcium signaling, reactive oxygen species, or nitric oxide signatures have been described, and hormonal regulation controls the establishment and functioning of mutualistic symbiosis (Pozo *et al.*, 2021). As a result of this mutualism, many beneficial endophytic fungi can help plants to take up and use soil nutrients, through bio-stimulation of many compounds and/or enhancement of their availability. These nutrients have been linked to the promotion of plant growth and development, thus increasing plant yields (Pozo *et al.*, 2021). Additionally, many endophytic fungi can improve plant resistance to pathogen and insect attacks (Xia *et al.*, 2019).

Entomopathogenic fungi are known to act as insect control agents, either through a plant-mediated response or by exerting a direct insecticidal effect. Several recent studies have revealed that entomopathogenic fungi, often considered specifically as insect pathogens, actually play extra roles in nature, such as endophytism, plant disease antagonism, plant growth promotion, rhizosphere colonization, and management of various abiotic stresses (Vidal and Jaber 2015). The entomopathogenic fungus *Beauveria bassiana* (Balsamo-Crivelli) Vuillemin (*Ascomycota*: *Hypocreales*) has been identified as a colonizing endophyte in a broad spectrum of plant host species, often promoting their growth and defense (Wei *et al.*, 2020; Barra-Bucarei *et al.*, 2019). Several *B. bassiana* bio-formulations are already commercially available for field control of plant pests, and others are under study to further improve fungal performance (Felizatti *et al.*, 2021). In tomato, the growth-promoting activity of *B. bassiana* has been related to increased nutrient bioavailability, and this could be partly explained by the production of iron siderophores and by the phosphate solubilization, without ruling out the role of phytohormones or growth regulators (Barra-Bucarei *et al.*, 2019). In terms of defense promotion, *B. bassiana* has been widely used to control various agricultural pests and vectors of human diseases, including mosquitoes, flies, and ticks (Chen *et al.*, 2017). Previous studies have provided evidence for a direct entomopathogenic effect of several *B. bassiana* strains against the whitefly *Trialeurodes vaporariorum* in tomato, best seen with the commercial *B. bassiana* strain ‘Naturalis’ (Oreste *et al.*, 2015). This strain has been selected as an efficient biocontrol agent also against the fungal pathogens *Botrytis cinerea* and *Alternaria alternata* and the aphid *Macrosiphum euphorbiae* (Sinno *et al.*, 2021). Several other studies have demonstrated the efficiency of *B. bassiana* colonization in helping several plant species to cope with attacks by pests and pathogens (Wagner and Lewis, 2000; Akello *et al.* 2008; Ownley *et al.* 2008; Akello and Sikora 2012; Jaber and Salem 2014). However, the effect of plant colonization by *B. bassiana* is still poorly understood at the molecular level. In particular, it is largely undisclosed how this colonization alters primary and secondary plant metabolism and enhances general defense responses. To date, only a few -omics studies have attempted to investigate *B. bassiana* symbiotic interactions at the host’s specific tissue or cellular and subcellular levels. Plant responses to *B. bassiana* were investigated in Arabidopsis at the transcriptomic and metabolic level and by analysing changes in defense-related phytohormones and glucosinolates. These studies showed evidence for transcriptional reprogramming of plant defense pathways, including those of genes encoding pathogenesis-related proteins, phytoalexin, jasmonic acid (JA) and salicylic acid (SA) signaling (Raad *et al.*, 2019). The use of proteomics and hormonomics could help in understanding better the molecular basis of the regulation of developmental and metabolic pathways during symbiotic interaction. Indeed, proteomics has recently been targeted as an excellent analytical tool for the in-depth understanding of molecular mechanisms underlying plant–microbe interaction (Jayaraman *et al.*, 2012; Khatabi *et al.*, 2019). Several proteomics studies addressing the characterization of pathogenic mechanisms of *B. bassiana* on insect hosts have been extensively and recently reviewed (Harith-Fadzilah *et al*, 2021). Nonetheless, to the best of our knowledge, only one study has investigated the effect of *B. bassiana* colonization on plant proteomes, highlighting the induction of proteins related to photosynthesis, energy metabolism, and defenses (Gómez-Vidal *et al.*, 2009). A large-scale hormone analysis has not yet been performed on plants colonized *by B. bassiana*, and hormonomics could facilitate the investigation of the hormone network involvement in the observed phenomenon (Šimura *et al.*, 2018).

In this work, we explored tomato leaf proteome changes over time induced by *B. bassiana* strain ‘Naturalis’. Proteomics identified molecular pathways affected by *B. bassiana* associated with primary and secondary metabolism and plant growth. Interestingly, early down-regulation of proteins linked to defense responses to fungus and up-regulation of calcium channel and transporter related proteins suggested a well-established plant–fungus symbiosis. Moreover, the enrichment of the molecular pathways linked to protein/amino acid turnover and to the biosynthesis of energy and defense compounds suggested possible strategies exploited by the plant to take full advantage of the beneficial interaction in enhancing growth, development, and response to stress. Hormone profile data showed up-regulation of different growth-related hormones, including gibberellins, as both precursors and active forms, and defense-related hormones like jasmonate. Finally, we demonstrated that *B. bassiana* is able to mitigate the oxidative stress induced in tomato plants by the necrotrophic fungus *B. cinerea.*

## Material and methods

### *Beauveria bassiana* fungal culture

*Beauveria bassiana* strain ‘Naturalis’ was cultured on potato dextrose agar (PDA) maintained at 25 ± 2 °C, and 14:10 h light–dark photoperiod for 20 d. Conidia were harvested by adding 5 ml of sterile distilled water to the sporulating cultures. The surface of the mycelium was gently scraped with a sterile L-spatula to remove spores. The liquid containing mycelia and spores was filtered through synthetic wool to remove the mycelium and collect the spore suspension in a sterile tube. The harvested conidia were shaken to obtain a homogeneous spore suspension. The concentration of the spores in the liquid inoculum was determined by a series of dilutions and counting the number of spores in a hemocytometer (Neubauer hemocytometer chamber) under a microscope. The spore concentration ranged from about 1 × 10^8^ to 10^9^ conidia ml^−1^ in approximately 5 ml volume. The spore suspension was stored at 4 °C until use.

### Plant growth and colonization by *Beauveria bassiana*

Tomato seeds (*Solanum lycopersicum* cv San Marzano nano, Top Seed, Sarno, Italy) were surface sterilized in 1% NaOCl for 5 min, then rinsed three times with sterile distilled water. Seeds were germinated on sterile filter paper (Sigma-Aldrich, St Louis, MO, USA) soaked with sterile distilled water, in the dark at 24 °C. Germination occurred in 4–5 d. Seedlings were individually transplanted to 110 ml pots containing commercial soil (Type 3 special Brill Soil, Georgsdorf, Germany). After 1 week, the emerged tomato seedlings (two-cotyledon stage) were watered with a variable volume of the *B. bassiana* conidial suspension to obtain a final concentration of 1 × 10^6^ conidia ml^−1^ of soil volume. Control plants were watered with sterile distilled water. The treatment was repeated after 2 weeks. To confirm endophytic colonization, tissue samples were collected and surface sterilized in 1% NaOCl for 3 min and rinsed three times with sterile distilled water. Each section was dried on sterile paper, then cut into six pieces (about 1 cm^2^), placed on 90 mm Petri dishes containing PDA supplied with 1% lactic acid to avoid bacterial contamination, and incubated at 25 °C in the dark. Plant pieces were monitored daily to determine if there was fungal growth emerging from the cut plant (white, cottony, dense hyphal growth). The fungal mycelia were isolated from the substrate close to the plant tissue and transferred to new dishes containing PDA, to obtain pure cultures for identification, which was carried out by examining the morphology of the fungal mycelia under a light microscope (Primostar 3, Zeiss Microscopy, Jena, Germany).

### Leaf proteome analysis

#### Plant material

Leaves of adult plants were harvested from two sets of plant samples: (i) root-colonized by *B. bassiana* and (ii) control plants (not colonized), at 5, 7, 12, and 19 d (T1, T2, T3, and T4, respectively) after the second colonization. For each time point, leaf area and stem height were measured with a ruler. Leaf area was calculated on the widest leaf with the following formula: leaf area=leaf length×leaf width. For the following proteome analysis, three biological replicates were used for each time point. In total, 24 samples were obtained.

#### Protein extraction

Sample preparation was performed following Bertini *et al.* (2019b). Briefly, powdered leaves were suspended in a lysis buffer comprising 10% trichloroacetic acid (TCA) in acetone and 10% dithiothreitol (DTT), and then kept at 20 °C for 2 h before centrifugation at 15 000 *g* for 14 min at 4 °C. Pellets were washed in acetone with 10 mM DTT, 2 mM EDTA, and 1 mM phenylmethylsulfonyl fluoride before being centrifuged again under the same conditions. The pellets were dried in a Speed Vac Concentrator (Savant, Thermo Fisher Scientific, Waltham, MA, USA). Pellets were lysed with 5 ml of 6 M urea in 100 mM Tris–HCl pH 8.0 in a Branson Digital Sonifier (1min cycle, 1 min run time, 20 s on, 10 s off, 30% amplitude). Samples were centrifuged at 4 °C, 15 min, 15 000 g and the pellet was discarded.

#### In-solution digestion

Samples (20 µg) were reduced with DTT (30 nmol, 37 °C, 60 min) and alkylated in the dark with iodoacetamide (60 nmol, 25 °C, 30 min). The resulting protein extract was first diluted to 2 M urea with 200 mM ammonium bicarbonate for digestion with endoproteinase LysC (1:100 w:w, 37 °C, o/n, Wako, cat. no. 129-02541), and then diluted 2-fold with 200 mM ammonium bicarbonate for trypsin digestion (1:100 w:w, 37 °C, 8 h, Promega cat. no. V5113). After digestion, peptide mix was acidified with formic acid and desalted with a MicroSpin C18 column (The Nest Group, Inc.) prior to LC-MS/MS analysis.

#### Chromatographic and mass spectrometric analysis

Samples were analysed using an Orbitrap Eclipse mass spectrometer (Thermo Fisher Scientific) coupled to an EASY-nLC 1200 (Thermo Fisher Scientific). Peptides were loaded directly onto the analytical column and were separated by reversed-phase chromatography using a 50-cm column with an inner diameter of 75 μm, packed with 2 μm C18 spectrometer particles (Thermo Fisher Scientific). The following buffers were used: 0.1% formic acid in water (buffer A) and 0.1% formic acid in 80% acetonitrile (buffer B). Chromatographic gradients started at 95% buffer A and 5% buffer B with a flow rate of 300 nl min^−1^ for 5 min and gradually increased to 25% buffer B and 75% buffer A in 79 min and then to 40% buffer B and 60% A in 11 min. After each analysis, the column was washed for 10 min with 100% buffer B. The mass spectrometer was operated in positive ionization mode with nanospray voltage set at 2.4 kV and source temperature at 305 °C. An Ultramark 1621 was used for external calibration of the Fourier-transform mass analyser prior the analyses, and an internal calibration was performed using the background polysiloxane ion signal at *m*/*z* 445.1200. The acquisition was performed in data-dependent acquisition mode and full MS scans with one micro scan at resolution of 120 000 were used over a mass range of *m*/*z* 350–1400 with detection in the Orbitrap mass analyser. Auto gain control (AGC) was set to 4E5 and charge state filtering disqualifying singly charged peptides was activated. In each cycle of data-dependent acquisition analysis, following each survey scan, the most intense ions above a threshold ion count of 10 000 were selected for fragmentation. The number of selected precursor ions for fragmentation was determined by the ‘Top Speed’ acquisition algorithm and a dynamic exclusion of 60 s. Fragment ion spectra were produced by high-energy collision dissociation at normalized collision energy of 28% and were acquired in the ion trap mass analyser. AGC was set to 2E4, and an isolation window of 0.7 *m*/*z* and a maximum injection time of 12 ms were used. All data were acquired with Xcalibur software v4.1.31.9. Digested bovine serum albumin (New England Biolabs cat. no. P8108S) was analysed between each sample to avoid sample carryover and to ensure instrument stability, and QCloud (Chiva *et al.*, 2018; Olivella *et al.*, 2021) was used to control instrument longitudinal performance during the project.

#### Data analysis

Acquired spectra were analysed using the Proteome Discoverer software suite (v2.3, Thermo Fisher Scientific) and the Mascot search engine (v2.6, Matrix Science; Perkins *et al.*, 1999). The data were searched against a Uniprot *Solanum lycopersicum* database (as in August 2020, 34 655 entries) plus a list of common contaminants (Beer *et al.*, 2017) and all the corresponding decoy entries. For peptide identification a precursor ion mass tolerance of 7 ppm was used for MS1 level, trypsin was chosen as enzyme, and up to three missed cleavages were allowed. The fragment ion mass tolerance was set to 0.5 Da for MS2 spectra. Oxidation of methionine and N-terminal protein acetylation were used as variable modifications and carbamidomethylation on cysteines was set as a fixed modification. The false discovery rate (FDR) in peptide identification was set to a maximum of 1%. Peptide quantification data were retrieved from the ‘Precursor ions quantifier’ node from Proteome Discoverer using 2 ppm mass tolerance for the peptide extracted ion current (XIC). The obtained values were used to calculate protein fold-changes and their corresponding adjusted *P*-values. The raw proteomics data have been deposited into the PRIDE repository (Vizcaíno *et al.*, 2015) and they are available via ProteomeXchange with the identifier PXD029422.

#### Bioinformatics analysis

All downstream data analysis and plotting were generated within the R environment, version 4.0.2 (R Foundation for Statistical Computing, Vienna, Austria). Evaluation of global proteome changes between treatments and across time points was performed by principal component analysis (PCA). To perform PCA, missing label-free quantification values for undetected proteins were imputed to 0. PCA was computed with the package *FactoMineR* version 2.4 (Lê *et al.*, 2008) on the log_2_-transformed matrix by applying an offset equal to the minimum value. Differences between PCA clusters were assessed by pairwise Mahlanobis distances with the R package *HDMD* version 1.2. Distances were calculated between the coordinates of the centroids of sample groups, on the principal components 1 and 2. Hotelling’s *T*^2^ test was applied to calculate significant differences between centroids distances with the R package *Hotelling* version 1.0-8. Patterns analysis of expression profiles across time points was performed by a hierarchical clustering analysis with the package *pheatmap* version 1.0.12 (Kolde, 2019). A joined matrix of the total unfiltered log_2_ fold change values resulting from the differential expression analysis of *B. bassiana* versus control was built and the fold change missing values of each time point derived from undetected proteins were imputed to 0. Hierarchical clustering was then calculated on rows (proteins IDs) by the complete-linkage clustering method on the Euclidean dissimilarity matrix. Gene ontology (GO) annotation of the *Solanum* proteome was retrieved from the EMBL-EBI Gene Ontology Annotation (GOA) proteome database (https://www.ebi.ac.uk/GOA/proteomes). The downloaded GOA database was then imported in the R environment and a custom database was built with the package *mgsa* version 1.36.0 (Bauer and Gagneur, 2021). Enrichment analysis was performed with the package *clusterProfiler* version 3.16.1 (Yu *et al.*, 2012) by hypergeometric distribution test. The background universe of the hypergeometric test was represented by a vector of length equal to the total number of detected proteins among all analysed conditions. GOs were considered significantly enriched when passing a *P*-value cutoff of 0.05 and the Benjamini–Hochberg *P*-value multiple testing correction. For the GOs, we used *clusterProfiler* to perform enrichment analysis of over-represented Kyoto Encyclopedia of Genes and Genomes (KEGG) pathways (Kanehisa *et al.*, 2017) on differentially expressed proteins (DEPs) at each different time point by the command enrichKEGG, which automatically retrieved the annotated pathways of *S. lycopersicum* (‘sly’) from the KEGG database (https://www.genome.jp/kegg/catalog/org_list.html). KEGG pathways were considered significantly enriched with a *P*-value <0.05 after Benjamini–Hochberg correction for multiple testing. For each time point, GO and KEGG enrichment analyses were performed on a filtered list of DEPs retaining only annotated proteins.

### Western blotting–SUnSET analysis

Western blotting–SUrface SEnsing of translation assay (WB-SUnSET) analysis was performed in leaves of plants root-colonized by *B. bassiana* 12 d after the second colonization (corresponding to the third time point) and in control plants (not colonized), as shown in ‘Plant material’. Leaf protoplasts were isolated according to Yoo *et al.* (2007). After incubation at 25 °C with 10 µg ml^−1^ puromycin for 60 min, protoplasts were lysed in 10 mM Tris–HCl buffer pH 7.6 containing 100 mM NaCl, 10 mM MgCl_2_, 0.1% Triton X-100, and 1 mM EDTA. The lysate was resuspended in Laemmli sample buffer, used for 15% (w/v) SDS-PAGE, and electrophoretically transferred onto nitrocellulose membrane for a WB-SUnSET assay. Protein markers were used to assess molecular masses (SMOBIO-PM 2610, SMOBIO Technology, Hsinchu City, Taiwan). Following electroblotting, the membrane was blocked for 30 min in phosphate-buffered saline (6.5 mM Na_2_HPO_4_, 1.5 mM KH_2_PO_4_, 3 mM KCl, 0.15 M NaCl, pH 7.4) containing 0.5% (v/v) Triton X-100 and 3% (w/v) BSA and then incubated overnight at room temperature with a mouse anti-puromycin monoclonal antibody (dilution 1:1000) as a primary antibody (MABE342, EMD Millipore Corp., Temecula, CA, USA). The immunoreactive bands were detected with horseradish peroxidase-conjugated goat-anti-mouse IgG (dilution 1:7000) as a secondary antibody using 4-chloro-1-naphthole as peroxidase substrate (Sigma-Aldrich). The same membrane was stained with Ponceau S (Advansta, Menlo Park, CA, USA) to normalize the protein loading.

### Phytohormone and related compound analysis

Analyses of plant compounds were performed in leaves of plants root-colonized by *B. bassiana* 19 d after the second colonization (corresponding to the fourth time point) and in control plants (not colonized), as in ‘Plant material’, according to the methods reported in Dziurka *et al.* (2016, 2022). About 20 mg of lyophilized and powdered plant material with a mixture of stable isotope-labeled plant hormone as internal standard were extracted three times with an organic solvent consisting of methanol: water: formic acid 15:4:1 (v/v/v). The three extracts were combined and then evaporated under N_2_. The dry extracts were resuspended in 3% methanol in 1 M formic acid and then cleaned on hybrid SPE cartridges (BondElut Plexa PCX, Agilent, USA) as previously described (Dziurka *et al.*, 2016). Qualitative and quantitative analyses of plant hormones and related compounds were performed on a HPLC-MS/MS system with UHPLC apparatus (Agilent Infinity 1260, Agilent, Germany) coupled to a triple quadruple mass spectrometer MS/MS (6410 Triple Quad LC/MS, Agilent, USA) equipped with electrospray ionization. Separation of plant hormones and related compounds was achieved on an Ascentis Express RP-Amide analytical column (2.7 μm, 2.1 mm×150 mm; Supelco, Bellefonte, PA, USA) at 60 °C, at linear gradient of water versus acetonitrile both with 0.01% formic acid. Gradient elution at flow rate of 0.5 ml min^−1^ was used. As internal standards the following were used: [^15^N_4_]dihydrozeatin (DHZ-N15), [^15^N_4_]kinetin (K-N15), [^2^H_5_]*trans*-zeatin riboside (t-ZR-D), [^2^H_5_]indole-3-acetic acid (IAA-D), [^2^H_4_]salicylic acid (SA-D), [^2^H_2_]gibberellin A_1_ (GA1-D), [^2^H_2_]gibberellin A_6_ (GA6-D), [^2^H_2_]gibberellin A_5_ (GA5-D), [^2^H_2_]gibberellin A_4_ (GA4-D), [^2^H_6_]*cis*,*trans*-abscisic (ABA-D), [^2^H_5_]benzoic acid (BeA-D), [^2^H_5_]jasmonic acid (JA-D5), and [^2^H_5_]dinor-12-oxo-phytodenoic acid (dinor-12-oxo-PDA-D). All standards were from OlChemim (Olomouc, Czech Republic), except JA-D which was supplied by CND Isotopes (Quebec, Canada), and dinor-12-oxo-PDA-D, which was supplied by Cayman Chemical Corp. (Ann Arbor, MI, USA), and were at the highest available purity, and all solvents were of LC-MS grade from Honeywell (Poznan, Poland). Multiple reactions monitoring transitions were used for the identification and quantification of all compounds of interest. Analyte quantification was based on calibration curves obtained with a pure standard of all compounds taking account of the recovery rates of internal standards used; details are given in Cioć *et al.* (2022). Physico-chemical determinations were performed in three biological replicates.

### Pathogen bioassay and oxidative stress analysis

#### Pathogen bioassay

Tomato plants were grown and colonized by *B. bassiana* as described in ‘Plant growth and colonization by *Beauveria bassiana*’. The infection with *Botrytis cinerea* was carried out as follows. *Botrytis cinerea* strain B05.10 (van Kan *et al*., 1997) was grown for 2 weeks on half-strength PDA (Difco Laboratories, Leeuwarden, the Netherlands) plates containing penicillin (100 μg ml^−1^) and streptomycin (200 μg ml^−1^) at room temperature. *Botrytis cinerea* spores were subsequently collected, filtered through glass wool, and resuspended in half-strength potato dextrose broth (PDB; Difco Laboratories, Leeuwarden, the Netherlands) to a final density of 1 × 10^5^ spores ml^−1^ and left 3 h under incubation. Four sets of plant samples were set up: (i) controls; (ii) *B. bassiana* colonized plants, treated with pure PDB droplets; and (iii) *B. cinerea* infected plants and (iv) *B. bassiana* colonized + *B. cinerea* infected plants, treated with droplets containing *B. cinerea* spores in PDB. Treatments (ii) and (iv) were carried out 19 d after the second colonization by *B. bassiana*. Treatments (ii), (iii), and (iv) were performed applying 5 μl droplets of the suspension to four leaves of each plant. All plants were placed under a lid increasing relative humidity to 100% to stimulate the infection. Three days after *B. cinerea* inoculation, lids were removed, and the symptoms were scored in five disease severity classes ranging from no symptoms (class I), non-spreading lesion (class II), spreading lesion (class III–class IV), up to severe spreading lesions with tissue maceration (class V) (Van Wees *et al*., 2013).

#### Reactive oxygen species detection

Reactive oxygen species (ROS) were detected according to methods available in previous studies (Proietti *et al*., 2019; Falconieri *et al.*, 2022), on the leaves of plant samples (i), (ii), (iii), and (iv) described above (‘Pathogen bioassay’). Briefly, ROS production was detected by using 2ʹ,7ʹ-dichlorofluorescein diacetate (DCFH_2_-DA; Sigma-Aldrich), which is oxidized to highly fluorescent dichlorofluorescein (DCF) when ROS are present. ROS were detected on 2 mm leaf sections cut around the spotted PDB/*B. cinerea* spores. A half leaf section was incubated at room temperature in 20 mM DCFH_2_-DA in 10 mM Tris–HCl solution (pH 7.4) for 45 min in the dark. As a negative technical control, the other half was incubated in 10 mM Tris–HCl (pH 7.4) only, under the same conditions. After the staining, samples were washed three times in 10 mM Tris–HCl (pH 7.4) for 10 min to remove the fluorophore excess and ultimately mounted on glass slides. Fluorescence was then observed under an LSM 710 confocal microscope (Zeiss Microscopy) with a Plan Neofluar 20/1.30 objective. Two laser excitations lines were used (488 nm for probe detection and 561 nm for chlorophyll autofluorescence). Quantification of green fluorescence detected by using 2ʹ,7ʹ-DCFH_2_-DA in all four tested conditions was performed with ImageJ, version 1.53s (NIH, Bethesda, MD, USA) as in Falconieri *et al.* (2022) and reported as the integrated density mean from three biological replicates.

#### Thiobarbituric acid reactive substance measurement

Measurement of thiobarbituric acid reactive substances (TBARS) was conducted on the leaves of plant samples (i), (ii), (iii), and (iv) described in ‘Pathogen bioassay’. Following the methodology described in Proietti *et al.* (2021), TBARS level was used as a measure of lipid peroxidation. In brief, 400 mg of frozen leaves was thinly ground using a mortar and pestle while liquid nitrogen was continuously added. The powder was resuspended in 3 ml of 0.1% TCA and vortexed until homogenized. Following a 10 min centrifugation at 15 000 *g*, 400 μl of the supernatant (or 0.1% TCA for the blank) was added to either 1 ml of 0.5% thiobarbituric acid (TBA) in 20% TCA (+TBA solution) or 1 ml of 20% TCA (−TBA solution) (dilution factor 1:3.5). The samples were incubated at 80 °C for 30 min before being cooled on ice. After 5 min of centrifugation at 15 000 *g*, the absorbance was measured at 532 nm, which represents the maximal absorbance of the TBA–TBARS complex, and at 600 nm to allow for non-specific turbidity adjustment. The molar extinction coefficient (ɛ_μM_) (0.155 μM^−1^ cm^−1^) of malondialdehyde (MDA), one of the primary products of membrane degradation, was used to determine the TBARS equivalent (nmol ml^−1^) as follows:


$$\begin{array}{l} {}[A/{{\;\varepsilon \;}_{\mu }}_{M}MDA]\;\times \;dilution\;factor \end{array}$$

where


$$\begin{array}{l} A=\left[A_{532}nm\left(+TBA_{sol}\right)-A_{600}nm\left(+TBA_{sol}\right)\right]-\\ \left[A_{532}nm\left(-TBA_{sol}\right)-A_{600}nm\left(-TBA_{sol}\right)\right] \end{array}$$

#### Reactive oxygen species scavenging enzymes assays

The enzymatic assays were conducted on the leaves of plant samples (i), (ii), (iii), and (iv) described in ‘Pathogen bioassay’. One gram of leaf was finely ground with a mortar and pestle in the presence of liquid nitrogen and further processed as in Proietti *et al.* (2021). The powder was resuspended in 5 ml of a cold extraction solution containing 50 mM sodium phosphate buffer (pH 7.5), 1 mM EDTA, 1% (w/v) polyvinylpyrrolidone, 3 mM DTT, and a cocktail of protease inhibitors (Complete ULTRA tablets, Roche, Basel, Switzerland). The homogenate was centrifuged at 9000 *g* for 15 min at 4 °C (Universal 32R, Hettich, Tuttlingen, Germany), and the supernatant was utilized for enzyme activity tests. According to Proietti *et al.* (2021), superoxide dismutase (SOD) and catalase (CAT) activities were measured. SOD activity was measured using the SOD determination kit (Sigma-Aldrich, Uppsala, Sweden). The SOD Assay Kit is based on the utilization of Dojindo’s water-soluble tetrazolium salt (2-(4-iodophenyl)-3-(4-nitrophenyl)-5-(2,4-disulfophenyl)-2*H* tetrazolium, monosodium salt), which when reduced with a superoxide anion forms a water-soluble formazan dye. The rate of decrease is linearly related to the activity of xanthine oxidase, which is inhibited by SOD. Because absorbance at 440 nm is related to formazan dye concentration, SOD activity may be measured as inhibition activity by measuring the reduction in color development at 440 nm. Thus, inhibition activity corresponds to the quantity of total protein extract required to suppress formazan formation by 50% (IC_50_). CAT activity was determined by measuring the reduction in absorbance at 240 nm at 25 °C caused by H_2_O_2_ decomposition (ɛ_mM_=0.0436 mM^−1^ cm^−1^). The reaction mixture (1 ml final volume) comprised 19 mM H_2_O_2_ in 50 mM potassium phosphate buffer (pH 7.0); the reaction was initiated by adding 50 μg of protein extract. The specific activity of CAT was measured in μmoles of hydrogen peroxide consumed per minute per mg of protein sample. Ascorbate peroxidase (APX) activity was measured according to Bertini *et al.* (2019a). The absorbance decrease at 290 nm due to ascorbate oxidation was used to calculate APX activity (ɛ_mM_=2.8 mM^−1^ cm^−1^). Reaction buffer contained 50 mM of potassium phosphate, 0.5 mM of ascorbate, 0.1 mM of H_2_O_2_, and 0.1 mM of EDTA (pH 7.6).

#### Thiobarbituric acid reactive substance and reactive oxygen species scavenging enzyme data processing

Analyses were performed using three biological replicates and, for any biological replicate, on three technical replicates. Reported in the graphs are the weighted average and the mean standard deviation (SD), following the method reported in Taylor (1997):


$$\begin{array}{l} Weighted\;average=\frac{X_{1}W_{1}+X_{2}W_{2}+X_{3}W_{3}}{W_{1}+W_{2}+W_{3}} \end{array}$$

Where *X*_1_ is the average of technical replicates of the first biological replicate, *W*_1_=1/SD^2^, *X*_2_ is the average of technical replicates of the second biological replicate, *W*_2_=1/SD^2^, *X*_3_ is the average of technical replicates of the third biological replicate, and *W*_3_=1/SD^2^.


$$\begin{array}{l} Mean\;standard\;deviation=\frac{1}{\sqrt{W_{1}+W_{2}+W_{3}\;}} \end{array}$$

## Results and discussion

Beneficial fungi are often associated with plant physiological and metabolic reprogramming that can promote growth and strengthen the defense barriers. Here, we aim to shed some light on the tomato–*B. bassiana* interaction. Successful fungal colonization was confirmed as described under ‘Plant growth and colonization by *Beauveria bassiana*’, and *B. bassiana* was re-isolated from roots, stems, and leaves as previously observed by Wei *et al.* (2020) (Supplementary Fig. S1). We then investigated the molecular events regulating the tomato–*B. bassiana* interaction at the leaf level.

### *Beauveria bassiana* induces plant growth and triggers plant proteome reprogramming over time

Beneficial effects of *B. bassiana* colonization on plant growth (stem height and leaf area) were observed at all time points analysed, with a greater effect at T4 (Supplementary Fig. S2).

Plant proteome changes induced over time by *B. bassiana* were investigated by a combination of high-throughput profiling proteomics techniques and bioinformatics tools, identifying proteins across the different samples as shown in Supplementary Table S1. We performed a principal component analysis (PCA) on log_2_-transformed quantification values of all detected proteins (6783 unique proteins) to understand global proteome changes induced by *B. bassiana* treatment along with all time points. A PCA plot (Supplementary Fig. S3) shows clear separation of the four protein clusters related to the analysed time points with largest distance between T1 and T4 and smallest distances between T2 and T3, and T1 and T3 (Supplementary Table S2). Different distributions of samples belonging to different time points may reflect the different physiological stages of the plants. However, within the same time point, comparisons between *B. bassiana*-treated plants and controls show a trend of continuous increase in distances between groups, over the time series (Supplementary Table S2).

**Fig. 1.**
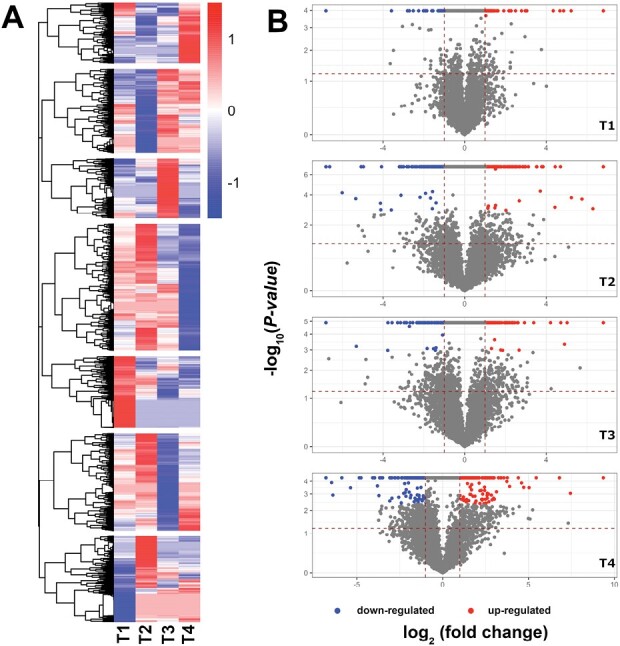
Proteome reprogramming after *B. bassiana* colonization. (A) Proteome-wide hierarchical clustering analysis at all time points. Red and blue colors represent *z*-scores of scaled fold changes of up- and down-regulated proteins of *B. bassiana*-treated plants versus control, respectively. The analysis highlighted the formation of seven clusters reflecting distinct expression patterns for each time point. (B) Volcano plots showing the results of the differential expression analysis for each time point. *x*-Axes display log_2_ fold change values comparing *B. bassiana*-treated plants versus control. Red and blue dots on the plot represent all significant up- and down-regulated proteins, respectively (FDR<0.05), after Benjamini–Hochberg test correction for multiple comparisons.

**Fig. 2.**
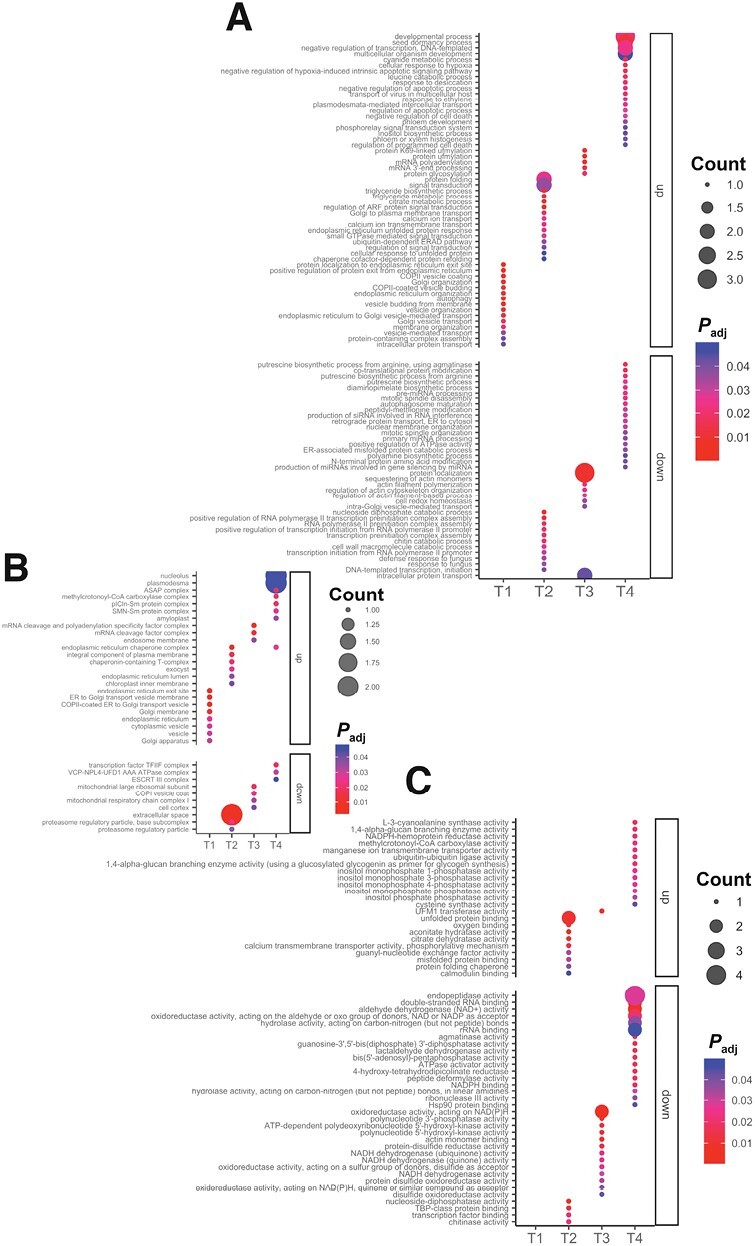
Dotplots of enriched GO terms over time. Different plots represent GO terms enriched for each ontological category. (A) Biological processes; (B) cellular component; (C) molecular function. Dots are colored with a red–blue gradient based on the *P*-values adjusted by the Benjamini-Hochberg test correction for multiple comparisons (*P*_adj_). Size of dots (counts) represents the number of DEPs up- or down-regulated that significantly enrich a specific GO term.

The PCA analysis results were confirmed and more precisely described with a proteome-wide comparative analysis of the protein abundance changes over time in both *B. bassiana*-colonized and control plants. Fold change (on a logarithmic scale) profiles between all detected proteins over all time points are summarized in a hierarchal clustering heatmap plot (Fig. 1A). This analysis shows that proteome reprogramming started in the initial phase of the root colonization by *B. bassiana* and continued until the last time point (Fig. 1A). A set of proteins with pronounced changes in abundance due to *B. bassiana* colonization was selected for each time point according to their significance (FDR adjusted *P-*value <0.05, *y*-axes) and a fold change (|log_2_ fold change| >1, *x-*axes) in comparison with the respective control (Fig. 1B). A total of 198 characterized proteins of interest over the four time points were identified. In particular, we detected 7, 30, 18, and 48 proteins up-regulated, with a log_2_ fold change value greater than 1, and 1, 18, 28, and 48 proteins underexpressed with a log_2_ fold change value less than −1, at T1, T2, T3, and T4, respectively. GO analysis (Yu *et al.*, 2012) of the selected proteins identified the biological processes (Fig. 2A), cellular component (Fig. 2B), and molecular function (Fig. 2C) enriched during the plant response to *B. bassiana* colonization over time, as further discussed below. Reported protein names are those from UniPROT.

For biological processes and cellular components, only up-regulated proteins were found at the first time point (T1). These were proteins related to protein transport from the endoplasmic reticulum to the Golgi such as ‘transport protein sec16’ (A0A3Q7HKB9) (Fig. 2A, B). The induction of this protein could be related to the entry mechanism of microbes into the host cell, which is preceded by the formation of a pre-infection structure at the host’s entry site, facilitating the targeting of vesicles towards the microbial entry site, especially in symbiotic fungi (Ivanov *et al.*, 2010). In T2, proteomic analysis revealed changes triggered by *B. bassiana* colonization in a consistent number of significant proteins involved in defense response, protein folding, and signal transduction (Fig. 2). Among the down-regulated processes, we found those involved in response to fungus and chitin catabolism. A marker of these down-regulated processes is the protein ‘acidic 26 kDa endochitinase’ (Q05539). Plants possess endochitinases with powerful antifungal activity and that have been reported as pathogenesis-related proteins induced by herbivores or phytopathogens (Neuhaus, 1999). In *Allium porrum* it has been reported that chitinases are down-regulated after the full establishment of symbiosis with the mycorrhizal fungus *Glomus versiforme* (Spanu *et al.*, 1989). Host penetration and colonization can elicit strong defense responses, and thus symbiotic fungi need to rewire the plant’s warning system to effectively suppress defense responses during the initial entry and proliferation phases (Shen *et al.*, 2018). It is tempting to hypothesize that the reduction of defense responses to the fungus observed at T2 reflects establishment of a symbiotic relationship in the early stages of interaction, rather than defense. This hypothesis is further corroborated by up-regulation of calcium ion (Ca^2+^) transport in which a ‘cyclic nucleotide-binding domain-containing protein’ (A0A3Q7HNZ3) is involved (Fig. 2). It has been suggested that the cyclic nucleotide gated channel (CNGC) is one of the most important calcium conduction channels. Several studies pointed out that Ca^2+^ flux across the plasma membrane is an early signaling step in establishing symbiosis and immunity, as documented in *Lotus japonica*, *Medicago troncatula*, and Arabidopsis (Zipfel and Oldroyd, 2017; Moscatiello *et al.*, 2018; Dietrich *et al.*, 2020; Yuan *et al.*, 2022).

While at the first two time points all protein changes probably reflect establishment of the plant–fungus symbiosis, in the last two time points growth and development-related processes were observed, such as up-regulation of processes involved in protein synthesis and folding or in providing energy to cells. For instance, the ‘methylcrotonyl coenzyme A (CoA) carboxylase complex’ and the ‘apoptosis- and splicing-associated protein (ASAP) complex’ were up-regulated at T4 (Fig. 2 B, C). ‘3-Methylcrotonyl-CoA carboxylase’ (MCC) (A0A3Q7ESD1) is an essential enzyme for the catabolism of the branched-chain amino acid leucine, which prevents the accumulation of toxic intermediates and provides energy to the cell (Tomassetti *et al.*, 2018). In most organisms, this complex leads to the formation of products controlling different metabolic pathways, such as the TCA cycle (Tomassetti *et al.*, 2018). Besides, the ASAP complex is involved in the regulation of mRNA processing and apoptosis. It binds to RNA in a sequence-independent manner and is recruited into the exon junction complex before or during the splicing process (Chen *et al.*, 2019). These two up-regulated processes suggest that, following the establishment of the plant–fungus symbiosis, there is a general reprogramming of the metabolic landscape towards energy production that can contribute to plant growth promotion. Among other T4 up-regulated proteins related to plant growth and development, there were those involved in ‘inositol monophosphatase activity’ (Fig. 2C), including ‘inositol-1-monophosphatase’ (A0A3Q7FZW9), involved in inositol synthesis. Inositol monophosphatase is developmentally regulated, with the highest levels detected in *Solanum lycopersicum* plant tissues undergoing rapid cell divisions (Gillaspy *et al.*, 1995).

Finally, among the up-regulated significant proteins, there was the ‘cysteine synthase’ (A0A3Q7IDI1) involved in ‘cysteine synthase activity’ (Fig. 2C). Sulfur is a macronutrient essential for plant growth and development. Sulfate, the most abundant form of sulfur in nature, is absorbed by plants and reduced and assimilated into cysteine (Wirtz and Hell, 2006). Cysteine is required to synthesize proteins and metabolites, being indispensable for growth and development. Cysteine also plays an essential role in plant immunity and redox signaling. It is involved in the detoxification of cyanide, which is essential for root hair development and plant response to pathogens, as demonstrated in Arabidopsis (Romero *et al.*, 2014).

**Fig. 3.**
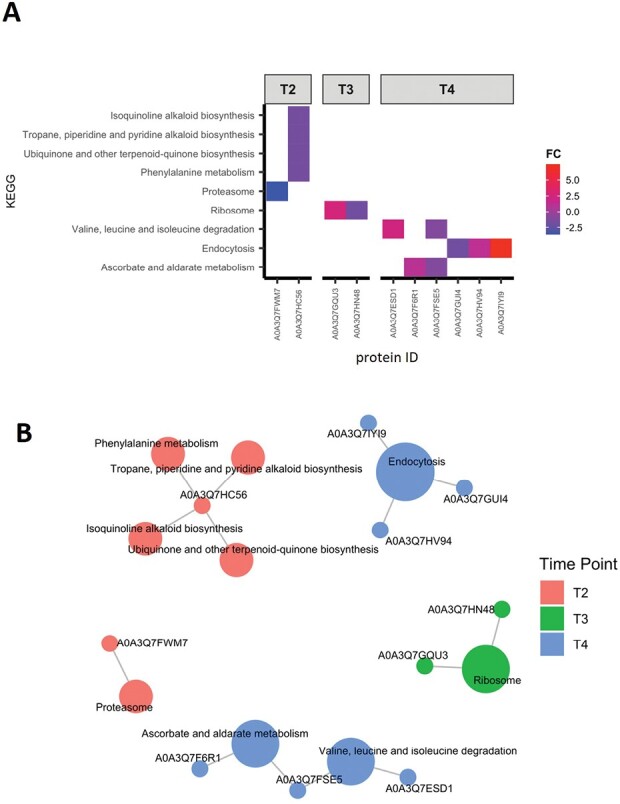
KEGG analysis over time. (A) Heatmap of significantly enriched KEGG pathways (*P*-values adjusted by the Benjamini–Hochberg test <0.05) at the different time points. Cells are colored by a red–blue gradient based on log_2_ of the fold change (FC) value of DEPs considering the ratio *B. bassiana*-colonized versus control plant for each time point. (B) Cnetplot of the enriched KEGG pathways for the different time points. The Cnetplot network graph highlights the contribution of one or more proteins to a KEGG pathway as well as the contribution of a single protein to multiple pathways.

On the other hand, at the later time points down-regulation of proteins involved in redox signaling suggests that *B. bassiana* does not generate oxidative stress levels harmful for the plant (Fig. 2C). To further deepen our understanding of the functions regulated by *B. bassiana* in tomato plants, we performed a KEGG pathways enrichment analysis on significant proteins (Fig. 3). It is noteworthy that since KEGG annotation is also based on orthologs, the results of this analysis are not be species-specific, and may rather be generalized (Kanehisa *et al*., 2015). KEGG analysis further revealed down-regulation of ‘aminotran_1_2 domain-containing’ (A0A3Q7HC56), a tyrosine aminotransferase at T2 (Fig. 3 A, B). Tyrosine aminotransferase enzymes have been implicated in diverting phenylalanine and tyrosine to secondary metabolite pathways, such as the production of antioxidants that scavenge free radicals and protect plants from various stresses (Parthasarathy *et al.*, 2018). In most plants, tyrosine aminotransferase also catalyses the transamination of tyrosine to 4-hydroxyphenylpyruvate, which serves as a precursor for the formation of numerous specialized metabolites with functions in plant defense (rosmarinic acid, dhurrin and benzylisoquinoline alkaloids), electron transport (plastoquinone and ubiquinone), and structural support (lignin) (Schenck and Maeda, 2018; Parthasarathy *et al.*, 2018; Xu *et al.*, 2019). It is noteworthy that plastoquinone and ubiquinone are two important prenylquinones, playing essential roles in the biosynthesis and metabolism of important chemical compounds involved in the stress response in several plant species, including tomato (Liu and Lu, 2016).

Moreover, at T2 we found the down-regulation of ‘AAA domain-containing protein’ (A0A3Q7FWM7), highly similar to the 26S Proteasome Regulatory Subunit 7 (source UniProt), included in the ‘proteasome’ term, and at T3, the up-regulation of ‘small subunit ribosomal protein S4e’ (A0A3Q7GQU3) included in the ‘ribosome’ term (Fig. 3A, B). The 26S proteasome regulatory subunit 7 is a subunit of the protease complex responsible for the degradation of a wide range of intracellular proteins, especially those modified with polyubiquitin chains (Yang *et al.*, 2004). Small subunit ribosomal protein S4e (also known as 40S ribosomal protein S4) is a structural constituent of ribosomes with protein and RNA binding activity. Ribosomal proteins play pivotal roles in translation efficiency and ribosome stability, and participate in important cellular processes, such as DNA repair, apoptosis, and regulation of gene expression, which are prominent for sustaining plant life (Song *et al.*, 2015). The down- and up-regulation of these two proteins, respectively, suggest that protein degradation is inhibited, with accumulation of proteins regulating growth and developmental pathways. Furthermore, Maspardin (A0A3Q7HV94), included in the ‘endocytosis’ pathway, was found to be up-regulated at T4 (Fig. 3A, B). Maspardin was first identified in humans, but has also been reported in plant species like sweet potato and rice (Liu *et al.*, 2014; Wang *et al.*, 2022). This protein is a member of the AB hydrolase superfamily, which has been found to localize in intracellular endosomal/*trans*-Golgi vesicles, and is likely involved in protein transport and sorting (Zeitlmann *et al.*, 2001). Accordingly, it has been reported that intracellular colonizers trigger massive vesicular membrane trafficking events (Leborgne-Castel and Bouhidel, 2014).

Taken together, these data indicate that plant–fungus symbiosis likely takes place at an early stage (T1 and T2), when defense-related processes are down-regulated. At the two last time points (T3 and T4), following a well-established plant–fungus relationship, processes related to vesicular trafficking, growth, and development are boosted, with a consequent beneficial outcome for plant health. To obtain a more comprehensive picture of the physiological rewiring at later time points, the impact of *B. bassiana* was further explored.

### *Beauveria bassiana* promotes protein synthesis

It is known that beneficial microbes have an important impact on biosynthetic pathways, particularly on protein synthesis, positively affecting plant health (Etalo *et al.*, 2018). During the symbiosis process, modification, degradation, and biosynthesis of peptides and proteins are critical for maintaining cell functions (Song *et al.*, 2015). Based on data reported in literature and on our results at T3, we analysed the impact of *B. bassiana* colonization on protein synthesis rate.

**Fig. 4.**
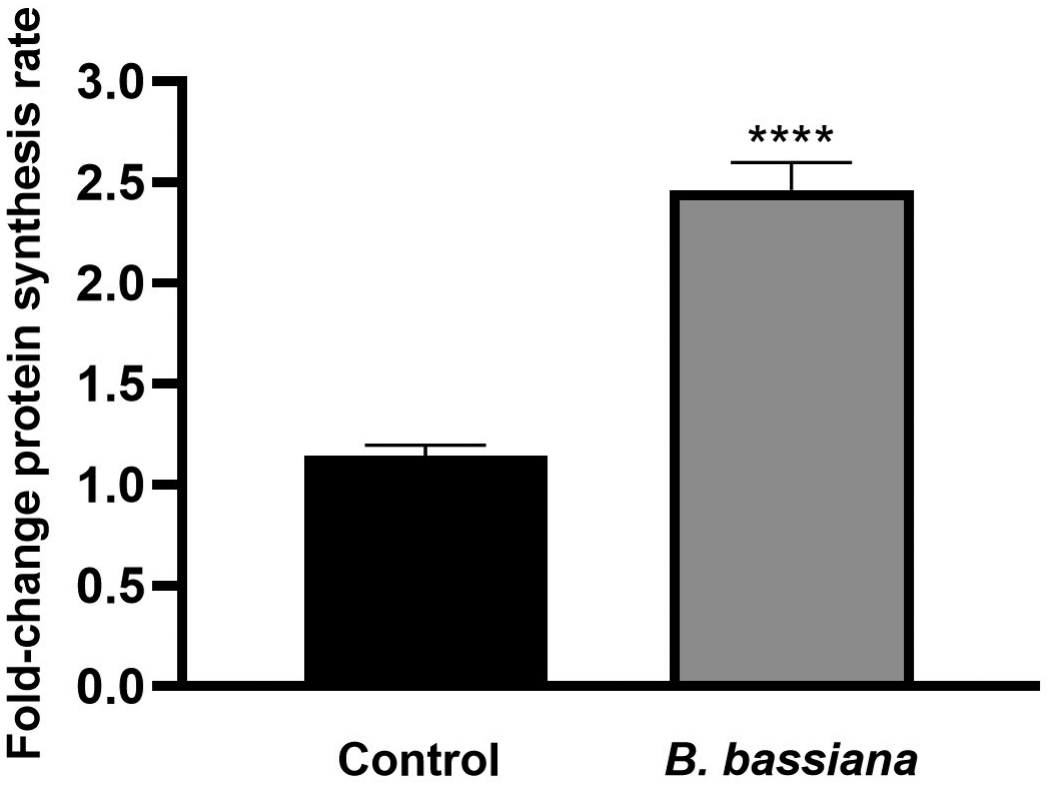
Protein synthesis rate of *B. bassiana*-colonized plants compared with control plants at T3, quantified using WB-SUnSET. The data shown are means of three biological replicates. Error bars represent SEM. Asterisks indicate the statistically significant difference between control and *B. bassiana*-colonized samples (*t*-test; ****P<0.0001).

A WB-SUnSET assay was conducted to assess the rate of protein synthesis in tomato leaf protoplasts isolated from *B. bassiana*-colonized and control plants at T3. The SUnSET assay is a non-radioactive method used to estimate the level of newly synthesized proteins. It has been widely used in animal cell research, but was only recently introduced, and is still little used, in plant cells assays (Van Hoewyk, 2016). This technique consists of treatment with the antibiotic puromycin, a structural analog of tyrosyl-tRNA. The incorporation of puromycin into nascent polypeptides causes translation termination and the formation of peptidyl-puromycin products (Van Hoewyk, 2016). Newly synthesized proteins containing puromycin can be measured by western-blot by using an anti-puromycin antibody. Here, the WB-SUnSET assay was optimized for performance on leaf protoplasts of tomato plants (Supplementary Fig. S4). The protein synthesis rate is calculated based on the intensity of the reference protein band in samples colonized by *B. bassiana* and not colonized, as in Supplementary Fig. S4. Fold-change is reported in Fig. 4. As shown, the rate significantly increased by double in *B. bassiana*-colonized plants compared with controls.

Our results confirmed that *B. bassiana* positively impacts the protein synthesis rate in the later stages of plant colonization. As a consequence, it can be hypothesized that an increase in protein biosynthesis induced by *B. bassiana* may contribute to improving plant health, providing energy and structural material for the living system.

### *Beauveria bassiana* affects the profile of plant hormone and related compounds

**Fig. 5.**
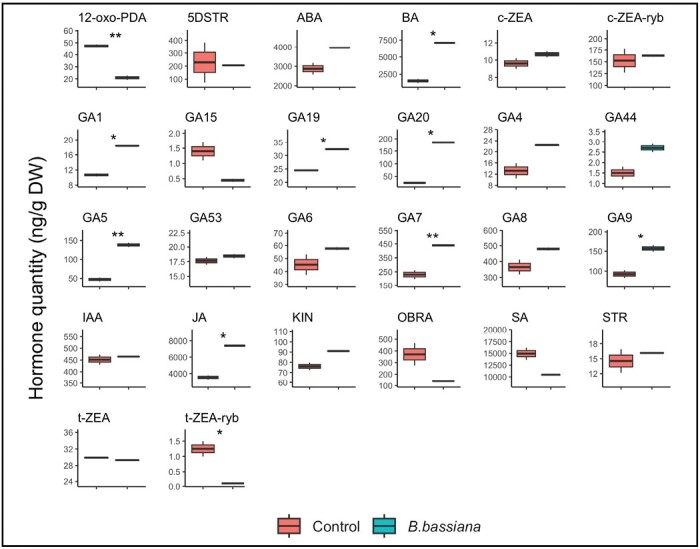
Hormones and related compounds identified by HPLC-MS/MS in plants colonized by *B. bassiana* and not colonized (control). Boxes represent the interquartile range (IQR) between the first and third quartiles, and the line inside represents the median (second quartile). Whiskers denote the lowest and the highest values within 1.5×IQR from the first and third quartiles, respectively. Asterisks indicate significant differences between *B. bassiana*-colonized vs control plants (*t*-test; Benjamini–Hockberg FDR correction; *P<0.05; **P<0.01). Physico-chemical determinations were performed in three biological replicates. 5DSTR, 5-deoxy-strigol; 12-oxo-PDA, 12-oxo-phytodienoic acid; ABA, abscisic acid; BA, benzoic acid; c-ZEA, *cis*-zeatin; c-ZEA-ryb, *cis*-zeatin riboside; GA1, gibberellin A1; GA4, gibberellin A4; GA5, gibberellin A5; GA6, gibberellin A6; GA7, gibberellin A7; GA8, gibberellin A8; GA9, gibberellin A9; GA15, gibberellin A15; GA19, gibberellin A19; GA20, gibberellin A20; GA44, gibberellin A44; GA53, gibberellin A53; IAA, indole-3-acetic acid; JA, jasmonic acid; KIN, kinetin; OBRA, orobanchol; SA, salicylic acid; STR, strigol; t-ZEA, *trans*-zeatin; t-ZEA-ryb, *trans*-zeatin riboside.

Plant hormones are involved in plant growth and development by inducing an array of cellular, morphological, and physiological processes (Weyers and Paterson, 2001). They are also involved in defense mechanisms against biotic and abiotic stresses (Aerts *et al.*, 2020; Waadt *et al.*, 2022). We performed analysis of leaf hormone and related compounds at T4, or 19 d after the second colonization of plants by *B. bassiana*. By using targeted HPLC-MS/MS we detected 26 hormones and related compounds that correlated to growth and defense. Among the detected compounds 10 were significantly affected by *B. bassiana* colonization with respect to controls (Fig. 5).

We observed up-regulation of growth-related hormones like gibberellin (GA) precursors and their active forms, as well as of related compounds with defense functions, such as benzoic acid and JA. GAs are a group of diterpenoid growth hormones strongly associated with growth promotion, including stem elongation and germination. The role of GAs in developing and maintaining plant-beneficial microbe symbioses is an emerging area of research (McGuiness *et al.*, 2019). GA1 and GA4 are the major bioactive GAs, and are more abundant than GA3 and GA7 in many plant species (Daviere and Achard, 2013). In our experimental conditions, we found several active GAs (GA1, GA5, GA7, GA19) as well as some GA precursors (GA9, GA20) significantly up-regulated in *B. bassiana*-colonized plants compared with controls. Some proteins that were up-regulated by *B. bassiana* colonization in our proteome dataset may be linked to the gibberellin pathway. Among them, NADPH-cytochrome P450 reductase (A0A3Q7H9M0) is the essential redox partner of multiple cytochrome P450s involved in the biosynthesis of a variety of secondary metabolites, including GAs (Bak *et al.*, 2011). Another *B. bassiana* up-regulated protein involved in gibberellin production, is the ‘C_2_H_2_-type domain-containing protein’ (A0A3Q7HYW7). This protein belongs to the histone deacetylase HD2 family, and recently emerged as a mediator of the switch from cell division to expansion in the root tip through repressing the transcription of GA2ox (Li *et al.*, 2017). The enzyme GA2ox2 catalyses the transition of GA9 and GA20 into further gibberellins (Israelsson *et al.*, 2005). This observation could explain the observed GA9 and GA20 accumulation following *B. bassiana* colonization.

An up-regulated hormone related compound involved in important plant development and survival processes is benzoic acid (BA). Among the key functions provided by BA and its derivatives are growth regulation, repellency, and pollinator attraction (Qualley *et al.*, 2012). BA has been documented to influence several physiological processes, including anthocyanin production, chlorophyll biosynthesis, ion uptake, root elongation, and enzyme activity (Maffei *et al.*, 1999). Additionally, Windisch *et al.* (2021) showed that lettuce roots secrete benzoic acid as a defensive metabolite against fungal pathogens. Taking all of the above data into account, an explanation for the accumulation of BA in our samples may be that this compound has a defensive role. Moreover, BA is the immediate precursor of SA (Chen *et al.*, 2009), and therefore an alternative hypothesis for BA accumulation is possible. However, in our dataset BA is up-regulated while SA is down-regulated. We might hypothesize that when plants recognize a colonizing microorganism, either a rhizobacterium or a fungus, as a biotrophic pathogen, they suddenly activate the SA pathway. This may have occurred in the early phases of *B. bassiana* colonization, and we may have missed the moment when SA peaked. Later, the microorganism responds by repressing SA by means of specific effectors to establish an interaction with the future host (Pozo *et al.*, 2015). Indeed, it has been reported that during a well-established symbiosis the SA pathway is repressed, the levels of JA and JA precursors increase, and there is an up-regulation of JA-responsive genes (Bastías *et al.*, 2021). The anticorrelation between SA and JA is often observed in dicots, rather than monocots, and in response to (a)biotic stresses (De Vleesschauwer *et al.*, 2013; Tamaoki *et al.*, 2013). This fine-tuned regulation of SA and JA signaling pathways by beneficial colonization could also play a role in increasing plant resistance to pests (Bastías *et al.*, 2021).

### *Beauveria bassiana* protects plants from *Botrytis cinerea*-induced oxidative stress

**Fig. 6.**
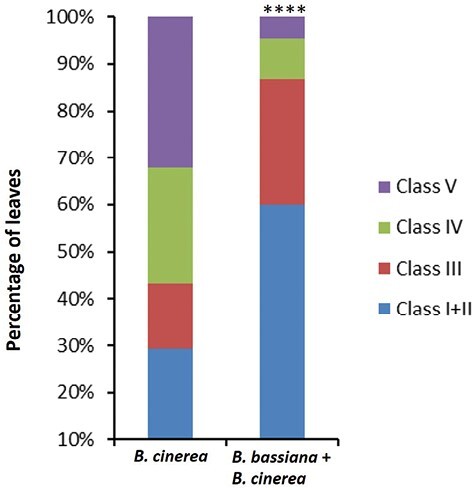
Distribution of disease symptoms of leaves from plants colonized by *B. bassiana* and infected with *B. cinerea* compared with *B. cinerea*-infected plants, 3 d after inoculation. The bars indicate the frequency distribution of disease symptoms. Disease rating is expressed as the fraction of leaves falling into five classes: I, only spot (no lesion); II, lesion smaller than 3 mm; III, lesion bigger than 5 mm; IV, lesion smaller than 1 cm; V, disease symptoms visible on the whole leaf. The percentage of leaves in each class was calculated per plant (*n*=10). Asterisks indicate statistically significant difference between *B. bassiana*+*B. cinerea* and *B. cinerea* samples (χ^2^ test; *P*<0.0001). Data shown are means of three biological replicates.

The fungal necrotrophic pathogen *B. cinerea* can affect all aboveground parts of tomato plants, both in the greenhouse and in the field. In recent years, many efforts have been made to develop an effective solution for the biological control of this disease in tomato (Bouaoud *et al.*, 2017; Minchev *et al.*, 2021). It is widely recognized that jasmonates are effective against necrotrophic pathogens (Antico *et al.*, 2012). The up-regulation of JA by *B. bassiana* in our dataset prompted us to investigate whether *B. bassiana-*colonized plants are more resistant to *B. cinerea* infection. Plants colonized with *B. bassiana* and subsequently infected with *B. cinerea* displayed less severe symptoms than plants infected with *B. cinerea* only (Fig. 6). It is well known that necrotrophic pathogens like *B. cinerea* can produce or induce plant production of ROS (López-Cruz *et al.*, 2016). Increased ROS levels in Arabidopsis and tomato enhanced *B. cinerea* colonization and necrotrophic development (Govrin and Levine, 2000; Kuzniak and Skłodowska, 2004). On the other hand, local ROS production enhanced resistance to *B. cinerea* in tomato (Asselbergh *et al.*, 2007). It has been recently pointed out that the endophyte-mediated plant antioxidant system may play an important role in alleviating stress damage by controlling ROS (Omoarelojie *et al.*, 2021; Sahu *et al.*, 2022). Additionally, arbuscular mycorrhizal colonization relieves oxidative stress induced by abiotic cues, monitored as a decrease in lipid peroxidation and activity of ROS scavenging enzymes (Molina *et al.*, 2020).

**Fig. 7.**
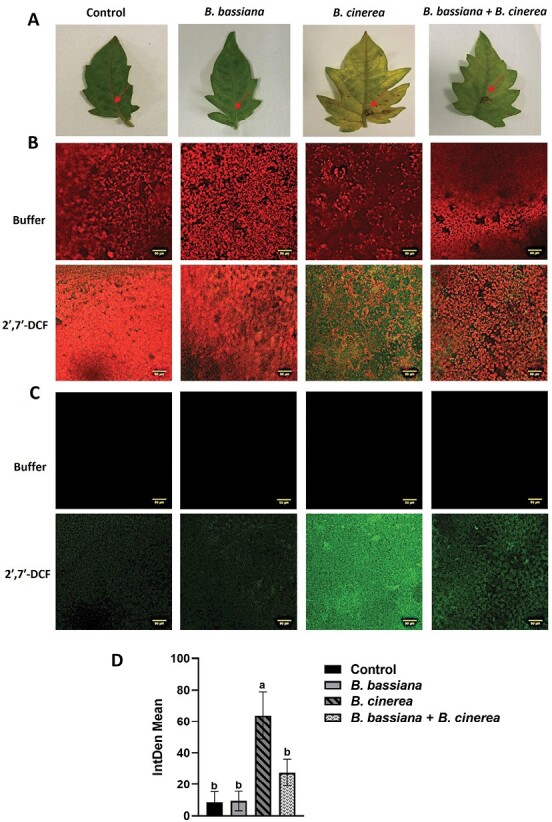
Detection of ROS in tomato leaves. (A) Representative leaves related to: (i) control, (ii) *B. bassiana* colonized plants, (iii) *B. cinerea* infected plants, and (iv) *B. bassiana* colonized + *B. cinerea* infected plants. Arrow indicates the site of inoculation of PDB droplet (i; ii) or *B. cinerea* droplet (iii; iv), further investigated for ROS assay. (B) Detection of ROS was carried out by using 2ʹ,7ʹ-DCFH_2_-DA or buffer (negative technical control). Fluorescence was observed under an LSM 710 confocal microscope with Plan Neofluar 20/1.30 objective. Laser excitations line was used, i.e. 488 nm for probe detection (green) and 561 nm for chlorophyll autofluorescence (red). Scale bar: 50 µm. Representative merged images are shown. (C) Green fluorescence only is shown. (D) Quantification of green fluorescence detected by using 2ʹ,7ʹ-DCFH_2_-DA in all four tested conditions, performed by ImageJ, version 1.53s. Reported is the integrated density mean from three biological replicates (one-way ANOVA, Tukey’s multiple comparison test: *P* <0.05).

To evaluate the impact of oxidative stress in response to *B. cinerea* infection in the presence or absence of *B. bassiana*, we measured ROS after treating plants with a ROS-sensitive dye (Proietti *et al.*, 2019; Falconieri *et al.*, 2022). The molecule DCFH_2_-DA diffuses into the cytoplasm through the plasma membrane and is deacetylated by intracellular esterase before being oxidized by ROS to form the green fluorescent dye 2ʹ,7ʹ-DCF. Originally, it was considered that H_2_O_2_ was the only ROS capable of converting DCFH_2_ to DCF, but recent studies showed that other ROS such as hydroxyl radical, hydroperoxides, and peroxynitrite may also oxidize DCFH_2_, albeit with considerably lower sensitivity than H_2_O_2_ (Proietti *et al.*, 2019; Falconieri *et al.*, 2022). Results revealed abundant green spots marking the presence of ROS in leaf samples infected by *B.cinerea* compared with those also colonized by *B. bassiana* (Fig. 7).

**Fig. 8.**
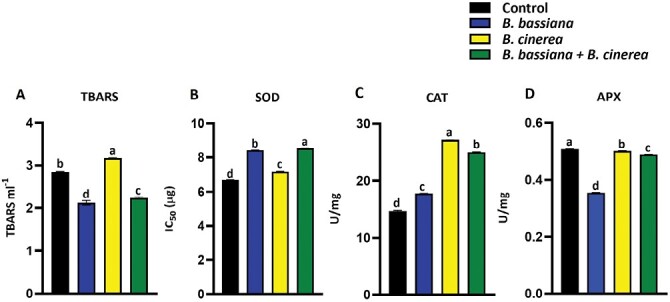
Lipid peroxidation and antioxidant enzyme activities in tomato leaves. (A) TBARS; (B) superoxide dismutase (SOD); (C) catalase (CAT); (D) ascorbate peroxidase (APX). Different letters indicate significant differences between treatments (one-way ANOVA, Tukey’s multiple comparison test: *P* <0.05). Data shown are means of three biological replicates.

Additionally, we measured lipid peroxidation (through the TBARS assay) and the activity of SOD, CAT, and APX enzymes (Fig. 8). Lipid peroxidation is a harmful process in plants, altering membrane characteristics, inducing protein breakdown, and reducing ion transport capacity, ultimately leading to cell death (Yamamoto *et al.*, 2001). One of the most commonly used methods to detect lipid peroxidation is the TBARS assay. Figure 8A shows that TBARS were significantly lower in *B. bassiana* + *B. cinerea* plants than in plants infected by *B. cinerea* only. This means that lipid peroxidation induced by the fungal pathogen was counteracted by the presence of the endophyte, reinforcing the idea of a protective effect exerted by *B. bassiana*.

The activities of SOD, CAT, and APX are frequently assayed as an indicator of oxidative stress in different living models (Marrocco *et al.*, 2017; Liu *et al.*, 2018). SODs are the initial line of defense against ROS within cells. These enzymes are members of the metal-enzyme family, which catalyses the dismutation of superoxide anions (O_2_^−^) into molecular oxygen (O_2_) and hydrogen peroxide (H_2_O_2_). Catalases typically convert two molecules of H_2_O_2_ to water and O_2_. APX is the other major H_2_O_2_-scavenging enzyme in plant cells, removing H_2_O_2_ via the ascorbate–glutathione cycle (Omoarelojie *et al.*, 2021). In our work, the IC_50_ of SOD in *B. bassiana* + *B. cinerea*-treated plants was higher than in *B. cinerea*-infected plant, implying that less protein extract is required to achieve 50% formazan inhibition. This suggested that SOD activity in *B. cinerea*-infected samples is higher than in *B. bassiana* + *B. cinerea*-treated plants (Fig. 8B). In our experiment, *B. cinerea* infection induced both CAT and APX activity, indicating that oxidative stress developed after pathogen attack. However, when infected plants are colonized by *B. bassiana*, APX and CAT activities decreased (Fig. 8C, D). It is thus plausible that *B. bassiana* implements molecular strategies to enhance resistance to *B. cinerea*, leading to slower pathogen fungal growth and less leaf area affected by the infection, in turn reducing the oxidative damage.

**Fig. 9.**
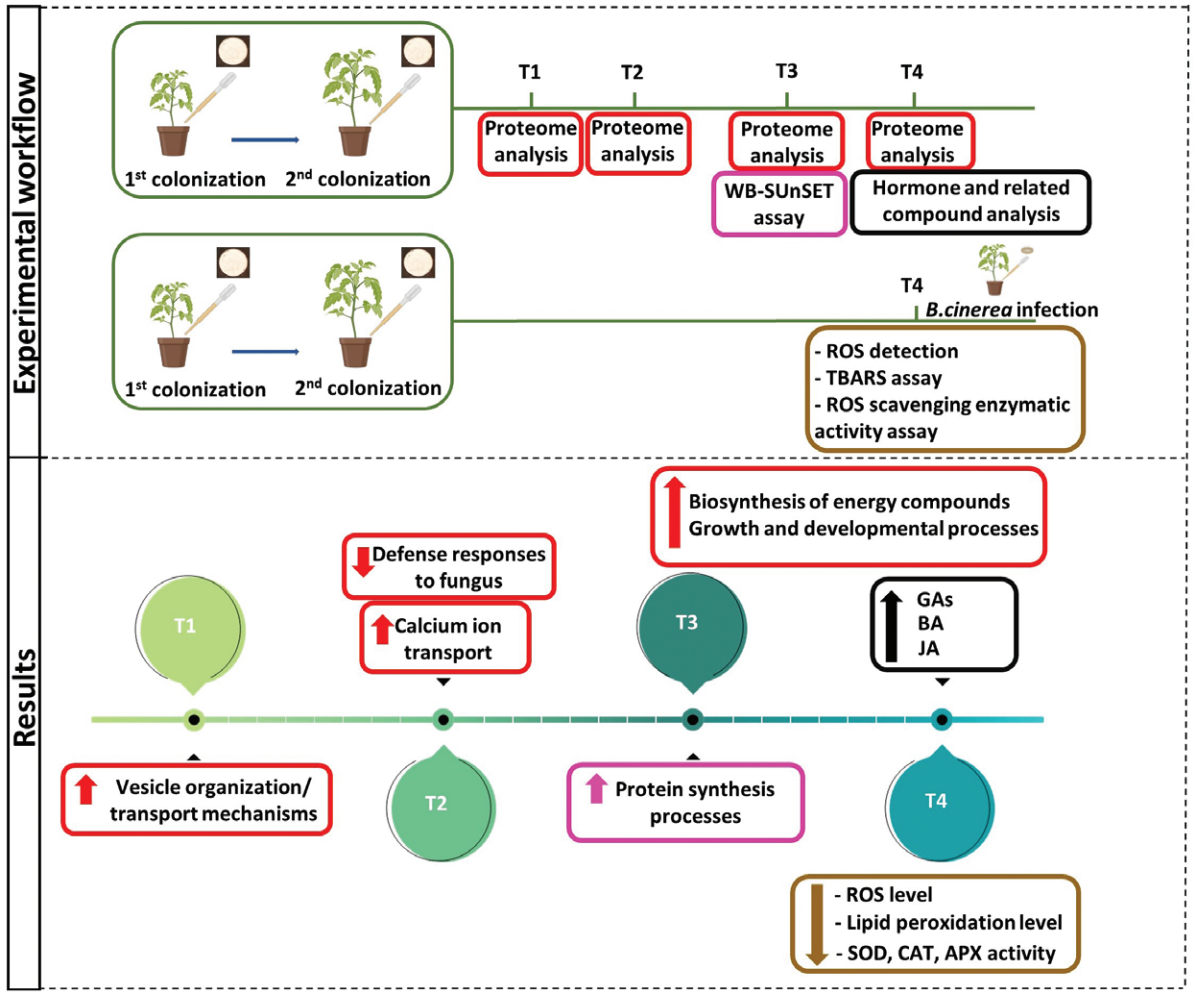
Summary of the experimental workflow and the main results obtained. In the experimental workflow box, the approaches used are distinguished by different colors. In the results box, the same color code is used. Arrows indicated up-regulated or down-regulated processes/compounds. Briefly, tomato plants have been double colonized by *B. bassiana* and a time course leaf proteome analysis was performed at 5, 7, 12, and 19 d after the second colonization (T1, T2, T3, and T4, respectively). Results highlighted up-regulation of vesicles organization (T1); down-regulation of defense response to fungus and up-regulation of calcium ion transport (T2); and up-regulation of biosynthesis of energy compounds and of growth and developmental processes (T4). Up-regulation of protein synthesis has been shown at T3 by a WB-SUnSET assay. Analysis of hormones and related compounds at T4 showed among others up-regulation of gibberellins (GAs), benzoic acid (BA), and jasmonic acid (JA). At T4, plants colonized by *B.bassiana* are more resistant to *B. cinerea* infection, likely impacting oxidative stress. Reactive oxygen species (ROS) detection, a thiobarbituric acid reactive compounds (TBARS) assay, and a ROS scavenging enzymatic activity assay showed a decreased ROS and lipid peroxidation level and decreased superoxide dismutase (SOD), catalase (CAT), and ascorbate peroxidase (APX) activities. Created with BioRender.com.

In conclusion, in this study, we detected key molecular pathways perturbed by *B. bassiana* colonization related to primary and secondary metabolism, likely responsible for a well-established tomato plant–fungus symbiosis and plant health promotion. Analysis of plant hormones and related compounds highlighted the impact of *B. bassiana* on growth- and defense-related substances. Finally, we showed that *B. bassiana* is effective against *B. cinerea* infection, reducing plant oxidative stress. A summary of the sequential activation of responses elicited in tomato plants colonized by *B. bassiana* is shown in the working model of Fig. 9. Future research will focus on dissecting the tripartite interaction *B. bassiana*–tomato–pathogen, which will increase our knowledge of the sophisticated way in which *B. bassiana* promotes plant health.

## Supplementary data

The following supplementary data are available at *JXB* online.

Fig. S1. Confirmation of *B. bassiana* endophytic colonization.

Fig. S2. Growth indexes of tomato plants colonized by *B. bassiana* or under control (not colonized) conditions.

Fig. S3. PCA plot showing the global proteome changes across all samples.

Fig. S4. Protein synthesis analysis by WB-SUnSET.

Tables S1. Proteins identified by LC-MS/MS across the different samples at the four time points (T1, T2, T3, T4).

Table S2. Summary of pairwise distances between clusters displayed on PCA plot (Supplementary Fig. S2).

erad148_suppl_Supplementary_Figures_S1-S4_Table_S2

erad148_suppl_Supplementary_Table_S1

## Data Availability

All data supporting the findings of this study are available within the paper and within its supplementary data published online. The mass spectrometry proteomics data have been deposited to the ProteomeXchange Consortium via the PRIDE partner repository with the dataset identifier PXD029422.
