# Supplementary material for: Beauveria bassiana rewires molecular mechanisms related to growth and defense in tomato
Source: J Exp Bot. 2023 Apr 24;74(14):4225–43. doi: 10.1093/jxb/erad148 (PMC10400115; doi:10.1093/jxb/erad148)
Supplement: erad148_suppl_Supplementary_Figures_S1-S4_Table_S2 [file erad148_suppl_supplementary_figures_s1-s4_table_s2.pdf]

Fig. S1

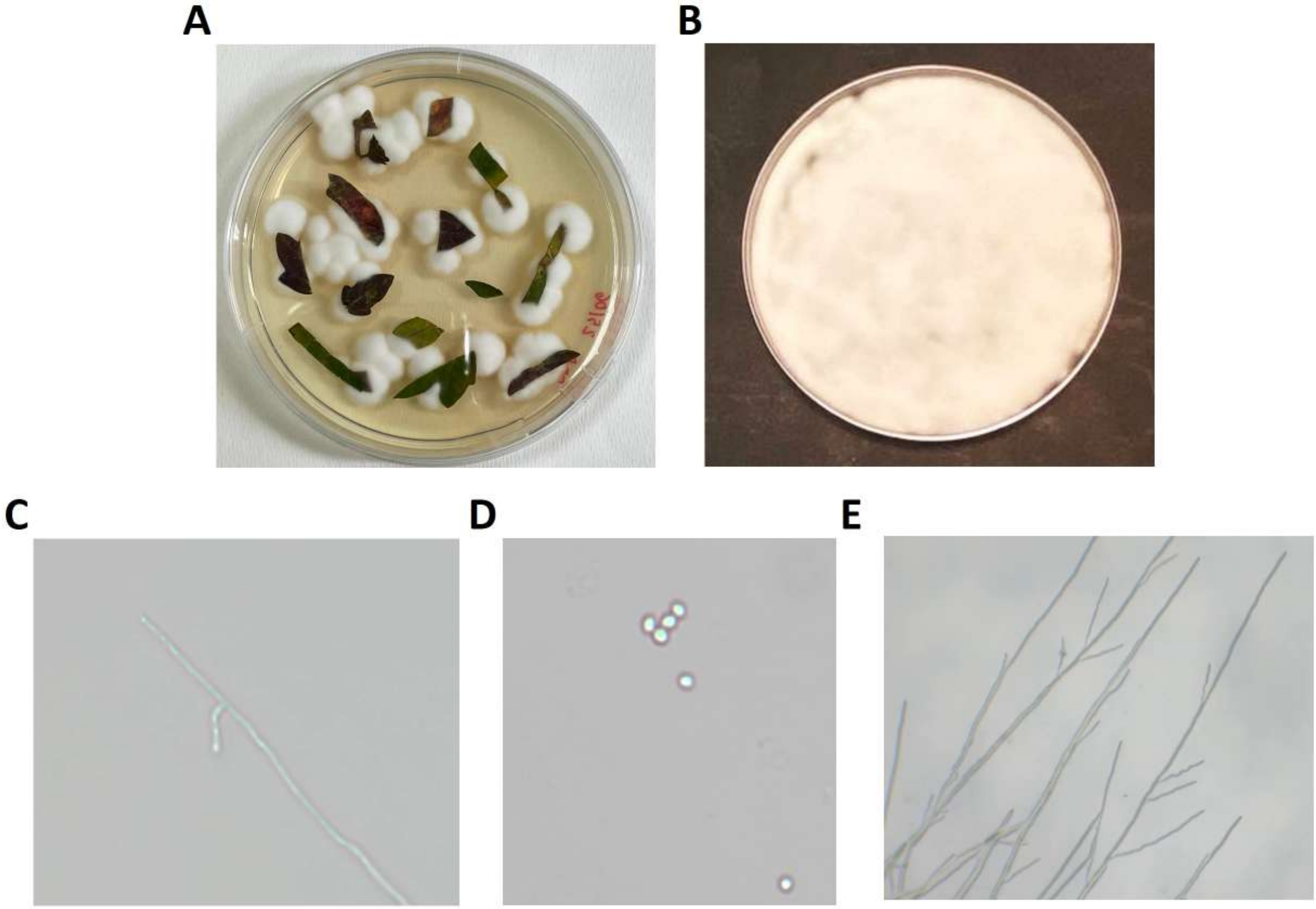

Supplemental Figure S1. Confirmation of *B. bassiana* endophytic colonization. (A) Fungal growth emerging from plant tissue sections. The fungal mycelia was isolated from the substrate close to the plant tissue and transferred to 90 mm Petri plates containing PDA and incubated at 25 °C in the dark. (B) Representative plate of *B. bassiana* pure culture. (C) Hyphae, (D) conidia and (E) mycelium of *B. bassiana* under light microscope (Zeiss, Primostar 3, 40x objective)

Fig. S2

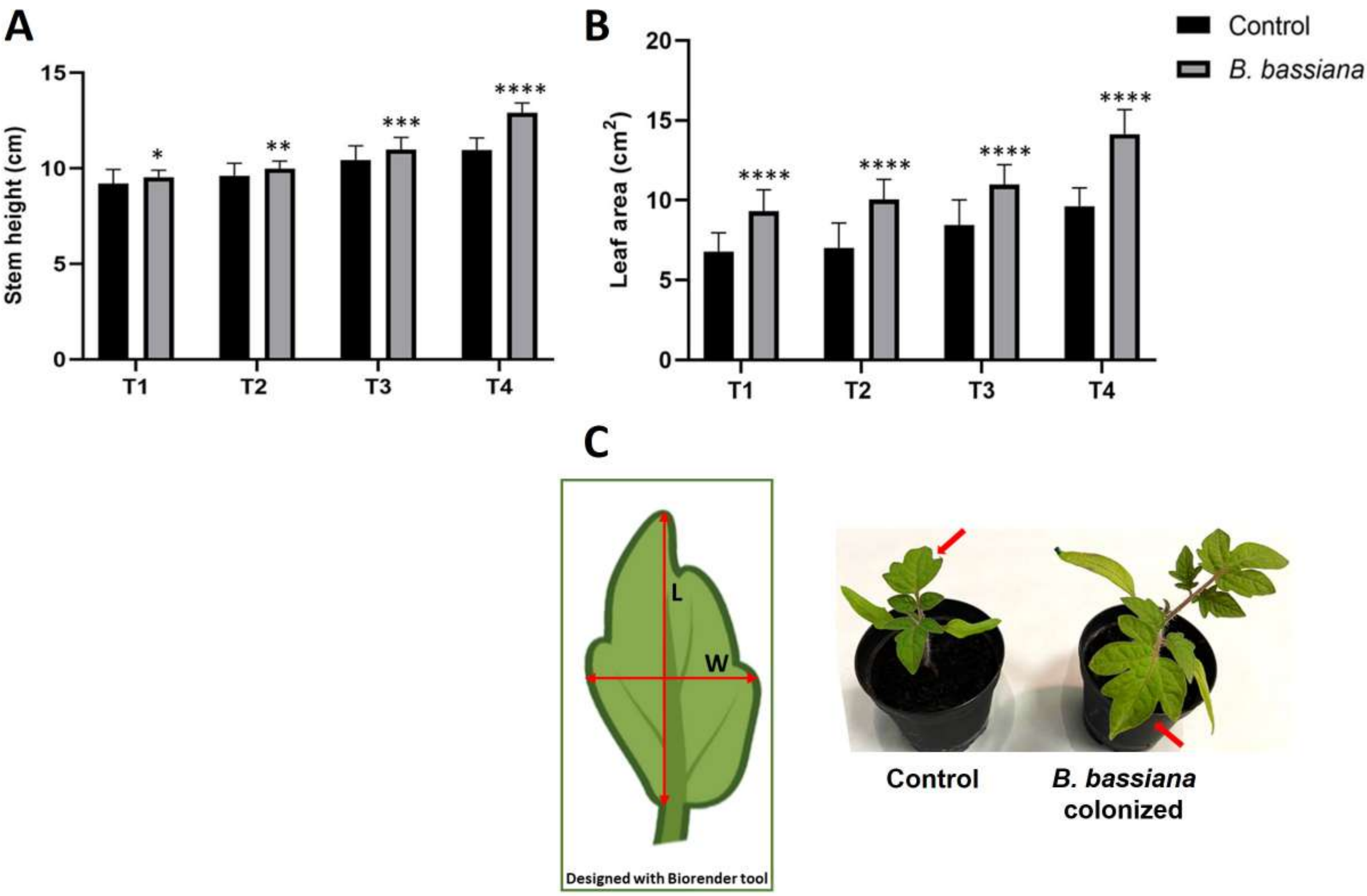

Supplemental Figure S2: Growth indexes of tomato plants colonized by B. bassiana or under control (not colonized) conditions. (A) Stem height. (B) leaf area (LA) measured in each considered time point. (C) LA was calculated on the widest leaf (indicated with the arrow) with the following formula: LA= L x W, where LA (leaf area); L (leaf length); W (leaf width).

Asterisks above the bars indicate significant differences between *B. bassiana*-colonized vs control plants (Two-way analysis of variance, Šídák's test; n=30; ****<0.0001; ***<0.0004,**<0.005, *<0.02).

Fig. S3

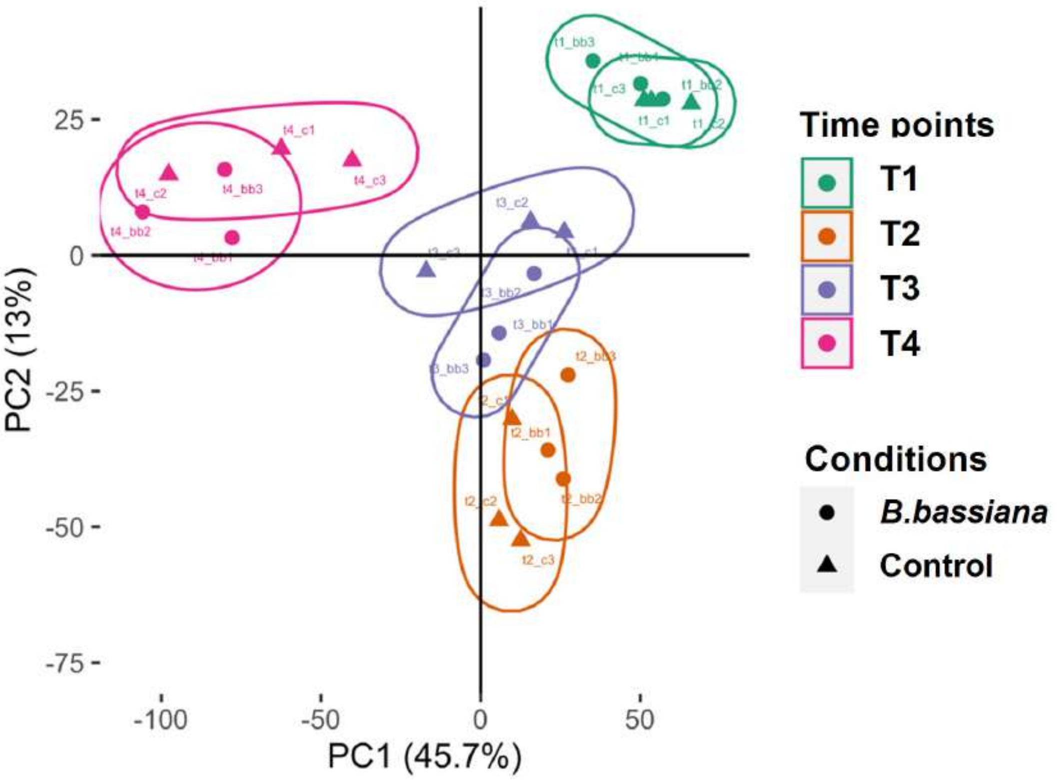

Supplemental Figure S3. PCA plot showing the global proteome changes across all samples. Convex hulls are drawn around sample groups. Samples belonging to control and *B. bassiana*-colonized plants are represented on the plot by triangles and circles, respectively.

Fig. S4

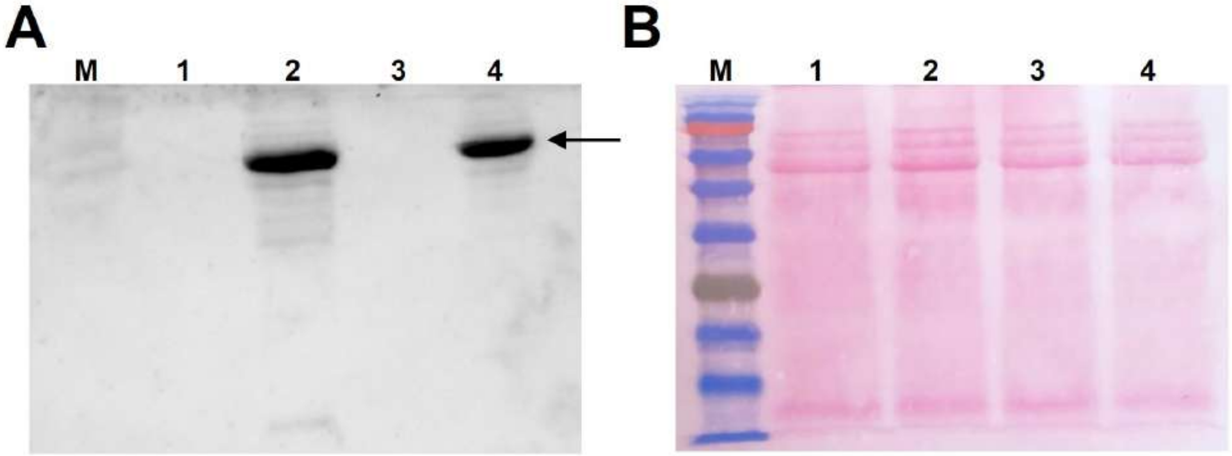

Supplemental Figure S4. Protein synthesis analysis by WB-SUnSET**. A:** Representative image of the WB-SUnSET of newly synthesized protein in *B. bassiana*-colonized tomato plant at 12 days after the second colonization and control (T3). Leaf protoplasts were treated or not with 10 µg ml^{-1} puromycin for 60min. Protein molecular weight markers (M), *B. bassiana* not treated (1) and treated with puromycin (2), control not treated (3) and treated with puromycin (4). Western blot was performed with anti-puromycin monoclonal antibody using control and *B. bassiana*-colonized total protein extracts (40 µg) from five-week-old plant leaves. **B**: Ponceau S staining was used to normalize protein loading. Fold-change of protein synthesis rate is given by the ratio of the protein band in lane 2 (*B. bassiana*-treated sample) and protein band in lane 4 (control sample) and normalized to the Ponceau stained band.

Supplementary Table S2. Summary of pairwise distances between clusters displayed on PCA plot (Supplementary Figure S2). Mahlanobis distances are calculated between each pairwise combination of time points and, withineach time point, between *B.bassiana*-colonized and control plants. Mahlanobis distance represents distancebetween centroids of each cluster calculated on principal component 1 (PC1) and principal component 2 (PC2). Hotelling T^2 test was applied to calculate statistical differences between multivariate means of samples amongclusters.

Pairwise Mahlanobis distance between clusters

between time points	distance measure	Hotelling's T^2 *p*value
T1-T2	7984.96	7.87E-07
T1-T3	1973.15	3.99E-05
T1-T4	61190.02	2.96E-06
T2-T3	1154.09	4.12E-04
T2-T4	12241.61	2.32E-07
T3-T4	8956.03	1.29E-05
**between conditions**	**distance measure**	**Hotelling's T^2 *p*value**
T1 c_vs_Bb	195.92	3.51E-01
T2 c_vs_Bb	245.49	3.78E-02
T3 c_vs_Bb	266.25	9.56E-02
T4 c_vs_Bb	452.77	2.98E-01
